# Drought trends projection under future climate change scenarios for Iran region

**DOI:** 10.1371/journal.pone.0290698

**Published:** 2023-11-09

**Authors:** Maryam Bayatavrkeshi, Monzur Alam Imteaz, Ozgur Kisi, Mohammad Farahani, Mohammad Ghabaei, Ahmed Mohammed Sami Al-Janabi, Bassim Mohammed Hashim, Baqer Al-Ramadan, Zaher Mundher Yaseen

**Affiliations:** 1 Department of Geography and Environmental Management, University of Waterloo, Waterloo, ON, Canada; 2 Civil and Construction Engineering, Swinburne University of Technology, Melbourne, VIC, Australia; 3 Department of Civil Engineering, Luebeck University of Applied Sciences, Lübeck, Germany; 4 Department of Civil Engineering, Ilia State University, Tbilisi, Georgia; 5 Department of Soil Science, Malayer University, Malayer, Iran; 6 Ministry of Energy, Iran Water Resources Management Co., Tehran, Iran; 7 Department of Civil Engineering, Cihan University-Erbil, Kurdistan Region, Iraq; 8 Environment, Water and Renewable Energy Directorate, Ministry of Science and Technology, Baghdad, Iraq; 9 Architecture & City Design Department, King Fahd University of Petroleum & Minerals, Dhahran, Saudi Arabia; 10 Interdisciplinary Research Center for Smart Mobility and Logistics, King Fahd University of Petroleum & Minerals, Dhahran, Saudi Arabia; 11 Civil and Environmental Engineering Department, King Fahd University of Petroleum & Minerals, Dhahran, Saudi Arabia; 12 Interdisciplinary Research Center for Membranes and Water Security, King Fahd University of Petroleum & Minerals, Dhahran, Saudi Arabia; Universiti Teknologi Malaysia, MALAYSIA

## Abstract

The study highlights the potential characteristics of droughts under future climate change scenarios. For this purpose, the changes in Standardized Precipitation Evapotranspiration Index (SPEI) under the A1B, A2, and B1 climate change scenarios in Iran were assessed. The daily weather data of 30 synoptic stations from 1992 to 2010 were analyzed. The HadCM3 statistical model in the LARS-WG was used to predict the future weather conditions between 2011 and 2112, for three 34-year periods; 2011–2045, 2046–2079, and 2080–2112. In regard to the findings, the upward trend of the potential evapotranspiration in parallel with the downward trend of the precipitation in the next 102 years in three scenarios to the base timescale was transparent. The frequency of the SPEI in the base month indicated that 17.02% of the studied months faced the drought. Considering the scenarios of climate change for three 34-year periods (i.e., 2011–2045, 2046–2079, and 2080–2112) the average percentages of potential drought occurrences for all the stations in the next three periods will be 8.89, 16.58, and 27.27 respectively under the B1 scenario. While the predicted values under the A1B scenario are 7.63, 12.66, and 35.08%respectively. The relevant findings under the A2 scenario are 6.73, 10.16, 40.8%. As a consequence, water shortage would be more serious in the third period of study under all three scenarios. The percentage of drought occurrence in the future years under the A2, B1, and A1B will be 19.23%, 17.74%, and 18.84%, respectively which confirms the worst condition under the A2 scenario. For all stations, the number of months with moderate drought was substantially more than severe and extreme droughts. Considering the A2 scenario as a high emission scenario, the analysis of SPEI frequency illustrated that the proportion of dry periods in regions with humid and cool climate is more than hot and warm climates; however, the duration of dry periods in warmer climates is longer than colder climates. Moreover, the temporal distribution of precipitation and potential evapotranspiration indicated that in a large number of stations, there is a significant difference between them in the middle months of the year, which justifies the importance of prudent water management in warm months.

## 1. Introduction

Drought as one of the natural disasters has enormous socio-economic consequences all over the world. Its long-lasting effects on agriculture, the ecosystem, and the economy over broad regions have been reported in several investigations [1, 2]. Since determining the exact dates of the beginnings and endings of droughts is never possible, estimating the effects and losses of drought is difficult. Furthermore, droughts could cause or accentuate other dangerous events such as floods, storms, and hurricanes [3]. This expensive phenomenon is driven by a variety of unpredictable factors which in turn enhances the complexity of this poorly understood phenomenon [4, 5]. On the other hand, climate change as a reaction of nature to human activities is projected will likely increase in the future due to the global warming effect, which deteriorates water shortage and threatens human lives and livelihoods [6]. Accordingly, climate change influences the ecosystem in terms of changing precipitation patterns and air temperatures, which may cause acute droughts. In the presented work [7], climate change would intensify natural disasters such as drought or flood. These consequences are visible in either short-term or long-term processes [8, 9].

Given that, identifying, and discovering changes in drought and finding their culprits are complicated. Moreover, high spatial and temporal variability of drought conditions results in uncertainty in forecasting drought. These circumstances necessitate the study of this natural phenomenon in every area which has been done in several investigations [10]. The climate change effect evaluation conducted on meteorological drought indices including Standard precipitation index (SPI) and Standardized precipitation-evapotranspiration index (SPEI) in Varamin plain [11]. Kamali and Asghari (2023) investigated the impact of future drought events under climate change conditions on groundwater storage in Najafabad aquifer in Iran [12]. Muthuvel et al. (2023) assessed global concurrent drought traits and their effects on maize yield under climate change in the USA, China, Brazil, Mexico, Argentina, France, and India [13]. The impacts of climate warming on the future hydrological droughts employed in 6,688 catchments by Gu et al. [14], and revealed that precipitation stress controls the development of historical droughts over ∼87% of global catchments. Predictability of meteorological drought in the semi-arid region based on the standardized precipitation index (SPI) was investigated by Elbeltagi, et al. [15]. An evaluation established for the future drought in the west of Iran by SPI under the A1B, B1 and A2 emission scenario of HadCM3 for the period 2011–2030 [10]. The result of their study indicated that the severity of drought will increase with longer return period. Quantifying the effect of climate change on characteristics of drought in Australia, Brazil, China, Ethiopia, India, Spain, Portugal and the USA showed that adaptation to drought risk will be vital in these regions for the first half of the twenty-first century [16]. Assessing features of future drought conditions over South Korea by [17] demonstrated an increase of 6% in frequency and severity of drought during the dry season. In the presented work [18], the authors investigated drought risk under climate and land use changes in the Frome catchment in the UK. In this survey, the drought indices predicted an increase in the severity of future drought events under the high emission scenarios. The study of [19] revealed an increasing and worsening drought event in the future than the last three decades for Northern Ethiopia. Monitoring drought patterns in the future climate highlighted the urgency for breeding drought-tolerant lines to secure crop productivity in rainfed regions such as Australia. Application of potential evapotranspiration (PET) in assessing influences of climate change on drought variation in the Andean high mountain basin illustrated that the evaluation of droughts in complex terrain in tropical regions should be promoted, where the representation of convection is the main limitation of global climate models [20]. In other work [15], the authors projected SPI and SPEI under adjusted outputs of global climate models in Turkey. The projections indicated fewer drought events for the near-future period of 2016–2040, with no potential extreme drought events. [21] compared indices of SPEI, SPI and Streamflow Drought Index (SDI) in identifying future drought characteristics in Cheongmicheon watershed, Korea, under general circulation models. According to the obtained results, the SPEI showed the most robust pattern with significant increases in both drought frequency and severity.

In regard to the previous investigations [22, 23], it can be inferred that drought characteristics alter over time due to climate changes and among regions with different climate conditions [24]. Accordingly, forecasting and monitoring drought would be worthwhile and beneficial in management of this phenomenon and would reduce losses due to it. Although several studies have addressed similar topics, it should be mentioned that the vast majority of them are based on regional scales for a short period of time. Whereas, the investigation of drought for a long time in large scales considering different climatic conditions under distinct climate change scenarios has been paid less attention. This kind of study is more benefit for an area with acute water shortage such as Iran which faces long-lasting droughts for several times. Given that, the present investigation could have an advantage in terms of considering distinct climates over a large scale. Moreover, evaluating drought using the SPEI for a long term in different climates can justify the importance of the present study. The findings of this investigation could be used in managing water resources and administering sufficient policies for tackling the future water shortage crisis.

## 2. Material and methods

**Fig 1:**
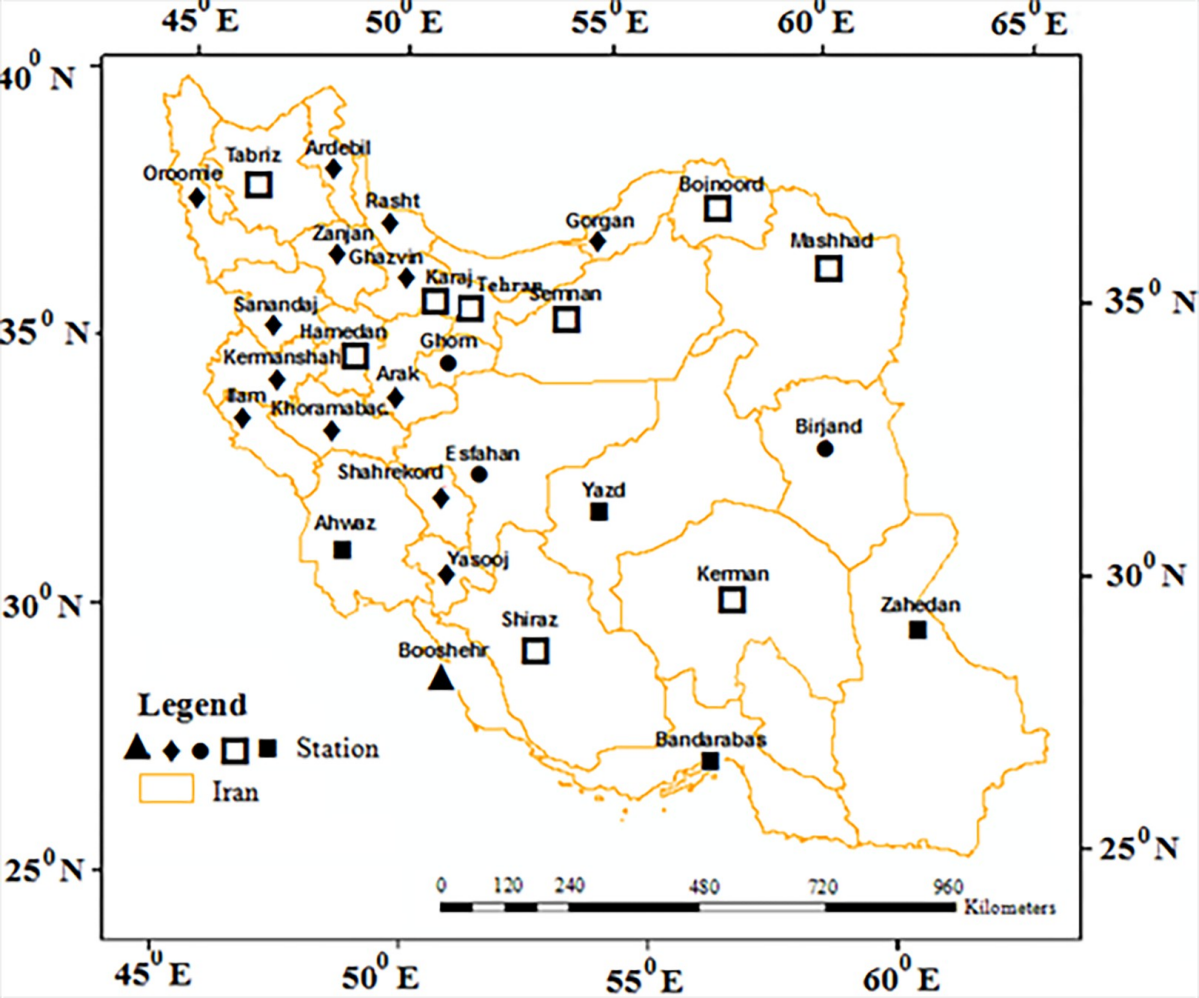
Geographical location of the selected stations in Iran. (▲: Tropical steppe: Semi-arid; hot, ◆: Interior Mediterranean: Mild winter; dry summer; hot summer, ●: Mid-latitude desert: Arid; cool or cold, □: Mid-latitude steppe: Semi-arid; cool or cold, ■: Tropical desert: Arid; hot).

Geographically, Iran is located at 25.05 to 39.78 Northern latitude and 44.08 to 63.30 Eastern longitudes. This study relied on the records of daily dataset collected from 30 synoptic stations for a 20 years period (1992–2010) for the assessment of the impacts of changes in climate conditions on drought. These stations were mainly considered for the quality of the data, as well as the uniformity in the period of data collection. Although some stations have data for a longer period of time, considering stations with the same time scale and no data missing matters to comparing the results under a reliable condition. There is no hesitation that any filling data technique has an error and will affect the findings, accordingly, to avoid any error related to missing data, this period of time was considered which had no data gap. In other words, datasets based on poor gauge coverage can produce substantial uncertainty when gaps are filled with data from different sources based on some climatology statistics [25]. Another consideration is that the stations are in different climatic locations which give a good sense of regional spatial distribution. Fig 1 depicts the distribution and location of the considered stations, as well as their climatic predispositions as per the Koppen system of climate classification [26].

Northern Iran is mainly the farming area of the country to the favourable weather condition in the area. This necessitated the citation of several weather stations in northern Iran than the other areas of the country. The assessment of drought can be carried out by a variety of indices, however accuracy of each index changes in different regions. For instant, high accuracy of the SPEI compared to other indices in different provinces of Iran has been reported by Zarei et al. [27]. The benefit of SPEI has been indicated in several investigations as well. Uddin et al (2020) mentioned that SPEI performs better than SPI because evaporative demand has a positive impact on defining drought conditions [28]. Wang et al. (2021) revealed that SPEI can be used to delineate spatial–temporal evolution of drought, drought characteristics, and impacts of drought at the regional and global scales which confirms the advantages of this drought index [29]. Accordingly, the SPEI was used in this study to evaluate drought conditions.

The determination of drought threats using the SPEI method was first introduced by [7] as a method that depends on two major parameters–temperature and precipitation. Being that temperature is the major variable in PET calculation, drought conditions are mainly determined by considering the variation between precipitation (P) and PET:

Di=Pi−PETi (1)

Estimating accumulated probabilities of *D*_*i*_ by probability density function is essential to calculate SPEI [30]. Due to negative values in *D*_*i*_, the probability density function of a three-parameter log-logistic is needed to calculate the SPEI as the following [7]:

$$f(x)=\frac{\beta }{\alpha }(\frac{x-\gamma }{\alpha }{)}^{\beta -1}{\left[1+{\left(\frac{x-\beta }{\alpha }\right)}^{\beta }\right]}^{-2}$$ (2)

where *α*, *β and γ* are scale, shape, and origin parameters, respectively, for D values (*γ*≻*D*≺∞).

The probability distribution function of the D series, according to the log-logistic distribution, is given by:

$$F(x)={\left[1+{\left(\frac{\alpha }{x-\gamma }\right)}^{\beta }\right]}^{-1}$$ (3)

The *F(x)*, values for the D series at different time scales adapt very well to the empirical *F(x)* values at the different observatories, independent to the climate characteristics and the time scale of the analysis.

With *F(x)*, the SPEI can easily be obtained as the standardized values of *F(x)*. For example, following the classical approximation of [31]:

$$SPEI=W-\frac{2.515517-0.802853W+0.010328W^{2}}{1+1.432788W+0.189269W^{2}+0.001308W^{3}}$$ (4)

$$where,\;W=\sqrt{-2\;\ln(p)}\;for\;p\leq 0.5$$ (5)

*P* presents the computed D value exceeding probability. *P = 1-F(x)*. if >0.5, then P is substituted by (1-p) and the sign of the resultant SPEI is reversed.

The SPEI mean and standard deviation values are zero and one, respectively. SPEI is an indication for standardized variable and thus it is comparable with other SPEI magnitudes over time and space. An SPEI of 0 signifies a corresponding value to 50% of the cumulative probability of D, this is according to a log-logistic distribution [32]. In the present investigation, the SPEI was run on package of the R 3.63 software.

Since the definition and conception of SPI is applied in the development of SPEI, different classes of drought intensity are based on recommended values by [33]. Table 1 reveals the classification of the drought categories with respect to SPEI.

**Table 1:** Different classes of drought intensity dependent on SPEI (Edwards and McKee, 1997).

Drought classes	Index value
Extreme wet	Index ≥.2
Severe wet	1.5≤index<2.0
Modest wet	1.0≤index<1.5
Normal	-0.99≤index<0.99
Moderate drought	-1.5<index≤1.0
Severe drought	-2.0<index≤-1.5
Extreme drought	index≤-2.0

**Fig 2:**
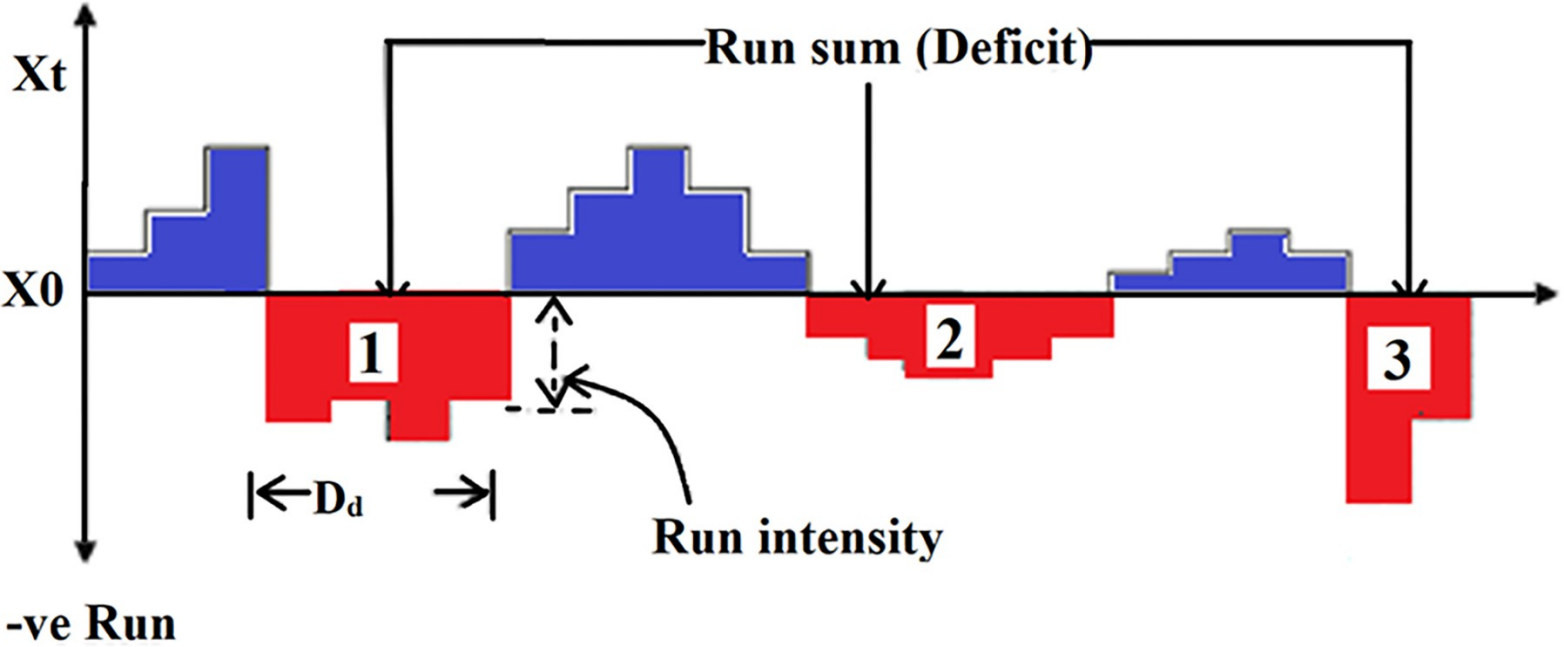
Description of drought characterization. 1: Drought with highest severity, 2: Drought with the longest duration, 3: Drought with the highest intensity.

For describing and analyzing the drought characteristics, the Run theory was used. In this theory, severity, duration, and intensity are considered as three important characteristics of drought. Fig 2 depicts these traits with *X*_*0*_ as threshold of *X*_*t*_ [34].

First, the climatic variables were computed by LARS-WG for the future period as this is needed for the calculation of future PET. LARS-WG was calibrated using maximum and minimum temperatures (Tmax and Tmin) and precipitation during base period of 1992–2012 for each station. By this way, these three variables (Tmax, Tmin and precipitation) were simulated for the future periods. For the calibration of LARS-WG, two comparison statistics, mean bias error (MBE) and normal root mean square error (NRMSE) were applied, which are defined as [35]:

$$NRMSE=\frac{{\left[\frac{1}{n}\sum_{i=1}^{n}{\left(X-Y\right)}^{2}\right]}^{0.5}}{\bar{X}}$$ (6)

$$MBE=\frac{\sum_{i=1}^{n}(X-Y)}{n}$$ (7)

where, *X* are the observed values and *Y* are the simulated values, $\bar{X}$and $\bar{Y}$ indicate the means of *X* and *Y* values respectively, *n* is the number of data.

After a good calibration of the developed model for the base period, the mentioned climatic variables were predicted for the future periods considering three scenarios, A1B, A2 and B1. The assumption made by the B1 scenario relies on an endurable world, that is, quick changes in economic developments, advancement of human rights equality and a consideration to environmental protection. This assumption considers that greenhouse gas emissions can be controlled and a pollutant controlling programme will be in place for the industries. The assumption made by the scenario of A1B relies on a wealthy world with a quick economic development (about 3percent per year), decrement in population (about 27% per year), quick technological development, convergence of culture and economy and a principle decrease in regional differences. The assumption made by the scenario of A2 relies on the assumption of a separate world. Different cultural characteristics in various areas of the world are expected to cause differences in the change of climate parameters [36]. Hence, the climatic variables were forecasted for three different periods (each having 34 years), 2011–2045, 2046–2079, and 2080–2112 under various scenarios.

In the second step, the amount of PET was calculated by the Hargreaves-Samani (HS) method. Due to the fact that the SPEI considers both precipitation and temperature, thus applying a method based on temperature can reflect the impact of temperature, which is completely transparent in the HS. Moreover, through comparing influence of climatic parameters on describing drought in Iran reported that temperature and precipitation parameters had the most influential effect on the fluctuation of drought severity in Iran [37]. Furthermore, previous works suggested this method in Iran [36, 38]. Therefore, PET was estimated by the HS equation which utilizes maximum and minimum temperatures, mean temperature and solar radiation as follows:

$$PET=0.0023R_{a}(T+17.8)\sqrt{TR}$$ (8)

where, PET is the potential evapotranspiration (*mm*), T is the mean air temperature (°C), TR is the difference between Tmax and Tmin (°C), and Ra is the extraterrestrial radiation (*MJm*^-2^*day* ^-1^). The Ref-ET software was implemented for estimating PET. Finally, considering the precipitation parameter during future years, the magnitude of the SPEIs were predicted for the future 102 years from 2011 to 2112 under each scenario.

For assessing the changes in the trend of SPEI between 2011 and 2112, the Mann-Kendall test was used. The Mann-Kendall test is applied to see whether Y values tend to decrease or increase with T (monotonic change). It can be hypothesized as the concentration independent of time of the chemical parameters [39] or H_0_: no correlation between x and y; Ha: x and y are correlated. To apply the test, Kendall’s S statistic is calculated from the Y, T data pairs:

S=P‐M (9)

where, *P* = the number of *Y*_*i*_<*Y*_*j*_ for all *i*<*j*, *M* = the number of *Y*_*i*_>*Y*_*j*_ for *i*<*j*:

$$\tau =\frac{s}{n(n-1)/2}\;for\;n>10$$ (10)

$$\begin{array}{l} \sigma_{s}=\sqrt{\left(n/18)\times (n-1)\times (2n+5\right)},\\ Z_{s}=\langle \begin{array}{c} \frac{S+1}{\sigma_{s}}\;if\;S<0\\ 0\;if\;S=0\\ \frac{S-1}{\sigma_{s}}\;if\;S>0 \end{array} \end{array}$$ (11)

Positive values of *Z* show upward trends while negative values of *Z* indicate downward trends.

## 3. Modeling results and analysis

Evaluating the accuracy of LARS-WG performance in simulating meteorological variables plays a key role in predicting weather parameters for the future periods. For this purpose, the consequences of statistical error indices in the base period are presented in Table 2.

**Table 2:** The results of LARS-WG test in prediction of weather variables after calibration in the base period.

	Precipitation		Tmin		Tmax	
Station	NRMSE	MBE	NRMSE	MBE	NRMSE	MBE
Ahvaz	3.90	-0.73	3.82	-0.71	0.03	-0.01
Arak	9.11	-2.32	0.56	0.04	0.33	0.07
Ardebil	7.03	-1.63	2.92	0.09	1.53	0.24
Bandar abas	4.37	0.61	0.38	0.03	0.49	0.16
Birjand	9.19	-1.19	0.70	0.06	0.73	0.18
Bojnoord	3.23	-0.7	0.57	-0.04	0.61	0.12
Boshehr	10.82	-2.46	0.19	0.04	0.70	0.21
Esfahan	14.37	-1.57	0.31	0.03	0.08	0.02
Ghazvin	2.98	0.76	0.01	0.01	0.23	0.05
Ghom	5.30	-0.69	0.58	-0.06	0.54	0.14
Gorgan	0.31	0.13	0.08	0.01	1.03	0.24
Hamedan	0.43	0.11	0.01	0.01	0.20	0.04
Ilam	11.26	-5.24	0.45	-0.05	0.49	0.11
Karaj	1.63	-0.34	0.33	0.03	0.01	0.01
Kerman	10.06	-1.09	0.67	-0.05	0.23	0.06
Kermanshah	3.04	-1.02	0.42	0.03	0.42	0.1
Khoram abad	11.11	-4.4	0.46	-0.04	0.43	0.11
Mashhad	0.19	-0.04	0.33	0.03	0.45	0.1
Oroomie	7.79	1.92	1.14	-0.06	0.67	0.12
Rasht	0.53	-0.56	0.57	-0.07	0.43	0.09
Sanandaj	3.13	-1	1.12	-0.07	0.27	0.06
Semnan	6.15	-0.73	1.02	-0.13	0.25	0.6
shahrekord	5.74	-1.57	2.93	-0.08	0.35	0.07
Shiraz	10.91	-3	0.63	-0.07	0.34	0.09
Tabriz	5.28	-1.05	1.53	0.12	0.63	0.12
Tehran	3.74	-0.74	0.15	0.02	0.60	0.14
Yasooj	9.43	-6.55	0.38	-0.03	0.39	0.09
Yazd	2.24	-0.10	0.39	-0.05	0.44	0.12
Zahedan	5.78	-0.37	1.38	0.15	0.81	0.22
Zanjan	6.17	-1.43	0.48	-0.02	0.71	0.13
Total	7.9	-1.233	1.4	-0.028	0.50	0.108

According to the information of Table 2, it can be inferred that the calibration of the model was successful with high accuracy. The values of NRMSE and MBE confirm the lowest error in simulating weather variables. Although there are significant differences among the values of NRMSE and MBE in few stations, in general, the results of Table 2 verify proper performance of the model in creating connection between observed and simulated values.

**Fig 3:**
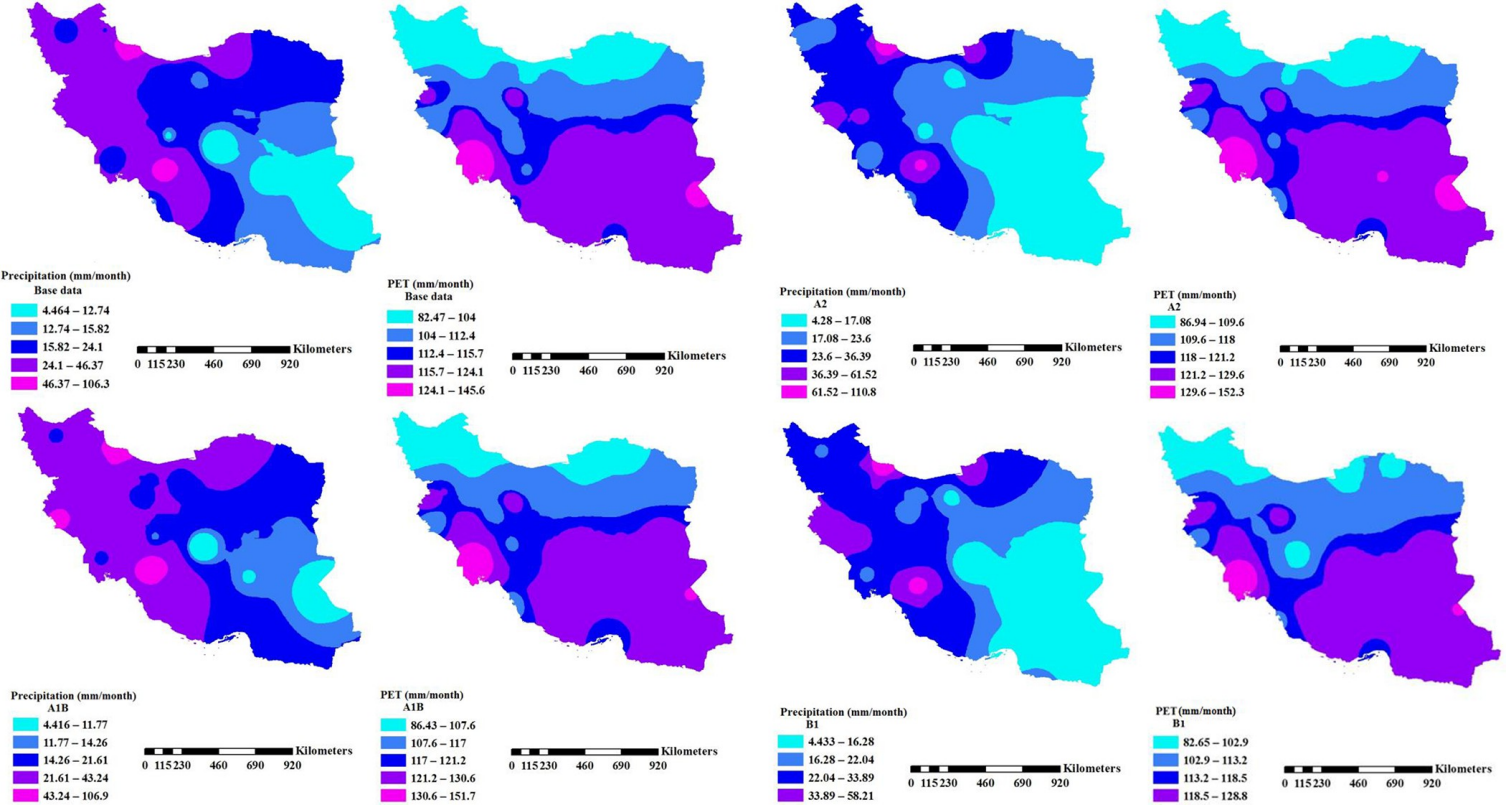
Spatial distribution of the monthly PET and precipitation in the base period and the future under the A1B, A2 and B1 scenarios.

Then, the prediction of the weather parameters for the upcoming period was done as earlier described in the materials and methods section. PET values were calculated using Eq 8 while the monthly spatial precipitation and PET distribution for the considered period and for up to the next 102 years (2011 to 2112) under different climate scenarios is shown in Fig 3. These figures were produced with ArcGIS 10.3 using the inverse distance weighted (IDW) procedure. Based on the assumptions of the IDW interpolation, features are indistinguishable if they are placed close to each other compared to the ones farther apart. The IDW strategy has been suggested to be used for data with a short range of variation [36]. It is obvious from Fig 3 that the Southern part of Iran has higher PET values (ranges from 124.1 to 145.6 mm/month) than the northern part (ranges from 82.47 to 104 mm/month).

Comparing the future PET under the scenarios indicate that the trend of spatial changes of PET under the A2 and A1B scenarios are similar to the PET in the base period, whereas there is a narrow margin between the PET changes under the B1 scenario and the base period. Nevertheless, the increasing trend of the PET in the three scenarios to the base timescale is transparent. These findings are reported by [36] that can confirm the current consequences. However, the spatial distribution of the precipitation pattern is completely the reverse of the PET, which is reasonably due to the identity of both the parameters. Contrary to the PET values, the lowest precipitation falls in the south-eastern parts of the country. This is noticeable that the category related to the highest precipitation under the A2 scenario is significantly less than other scenarios. In other words, the vast area of the country will receive precipitation less than 23.6 mm per month. However, this condition under the A1B and B1 scenarios seem better. Reduction of future precipitation in Iran has been reported by [40–42], that are in line with the current results.

Table 3 depicts the drought characteristics of each station in the base period. Describing drought was based on the recommendation of Edwards and McKee (1997) given in Table 1. The information of this table shows the percentage of dry and wet periods in the base period. For instance, value 1.32 percent in Ahvaz refers to the fact that the SPEI in 3 months out of 228 months (19 years) are less than -2, which refers to an extreme drought (Table 1). Overall, 17.02 percent of the studied months in the base period faced the drought, whereas 17.84 percent were in the wet condition. The most intensive drought was observed in Boshehr, by 439 percent. The highest number of dry months belonged to Birjand with 25.44 percent. It is noticeable that regions with high precipitation like Rasht or Gorgan have also experienced drought periods. The similar results are reported by [37], that support the present findings.

**Table 3:** Frequency of the SPEI based on the percentage of months in the base period.

Station	Extreme wet	Severe wet	Moderate wet	Normal	Moderate drought	Severe drought	Extreme drought
Ahvaz	0.00	11.40	3.95	72.37	3.07	7.89	1.32
Arak	0.44	5.26	14.91	64.91	6.58	6.14	1.75
Ardebil	3.07	3.51	8.77	65.79	14.04	3.51	1.32
Bandar abas	0.00	9.65	14.91	60.53	11.40	3.51	0.00
Birjand	0.00	2.19	21.93	50.44	23.68	1.75	0.00
Bojnoord	0.00	8.77	7.89	66.67	10.53	6.14	0.00
Boshehr	2.63	6.58	3.51	74.12	7.89	0.88	4.39
Esfahan	0.00	7.02	11.40	63.60	12.28	5.26	0.44
Ghazvin	0.00	8.33	10.09	64.47	10.96	5.70	0.44
Ghom	0.44	6.58	9.65	64.47	13.60	4.82	0.44
Gorgan	1.32	3.51	11.40	67.54	7.46	7.46	1.32
Hamedan	1.32	6.14	7.89	68.86	8.77	5.70	1.32
Ilam	0.00	7.89	7.89	67.11	11.40	5.70	0.00
Karaj	0.00	5.26	11.84	63.60	14.47	4.82	0.00
Kerman	0.00	9.21	9.65	64.91	11.40	4.82	0.00
Kermanshah	0.00	8.77	8.33	66.67	7.89	7.89	0.44
Khoram abad	0.44	7.02	11.40	64.47	8.77	7.02	0.88
Mashhad	0.00	9.65	6.58	67.54	9.65	6.14	0.44
Oroomie	0.00	10.53	10.09	61.84	14.47	2.19	0.88
Rasht	0.44	7.89	8.77	68.86	7.89	4.39	1.75
Sanandaj	0.44	6.58	11.84	62.28	10.96	7.89	0.00
Semnan	0.00	9.65	7.89	69.30	7.46	1.32	4.39
shahrekord	0.00	7.02	8.77	64.91	14.04	5.26	0.00
Shiraz	0.00	9.65	10.53	64.91	8.33	5.70	0.88
Tabriz	0.00	8.33	9.65	67.98	8.33	3.95	1.75
Tehran	0.00	4.82	11.40	61.84	17.54	3.95	0.44
Yasooj	0.00	3.07	14.04	63.16	13.60	5.26	0.88
Yazd	0.00	7.89	12.72	61.84	12.28	4.82	0.44
Zahedan	3.07	2.19	10.96	64.47	14.47	4.82	0.00
Zanjan	0.88	6.14	11.40	64.91	8.77	7.02	0.88
Total	0.48	7.02	10.34	65.15	11.07	5.06	0.89

For presenting the calculated SPEI values for the future period, the percentage of months that SPEI values are within each category to all the considered months (e.g., 102 years or 1224 months) was counted. The findings under each scenario are shown in Tables 4, 5, and 6. In these tables, the number of months in each category has been presented as a percentage for each studied station.

**Table 4:** Frequency of the SPEI based on the percentage of months under the B1 scenario.

Station	Period	Extreme wet	Severe wet	Moderate wet	Normal	Moderate drought	Severe drought	Extreme drought
Ahvaz	2011–2044	2.21	6.86	11.76	71.08	6.37	1.72	0.00
	2045–2078	1.96	7.60	10.78	71.57	6.13	1.96	0.00
	2079–2112	0.98	4.41	3.68	52.94	20.34	16.18	1.47
Arak	2011–2044	2.94	9.31	12.99	65.20	8.33	1.23	0.00
	2045–2078	0.49	5.15	8.82	67.89	14.22	3.43	0.00
	2079–2112	0.25	5.39	8.58	55.15	21.08	8.09	1.47
Ardebil	2011–2044	4.17	7.11	13.48	66.42	6.86	1.96	0.00
	2045–2078	1.47	5.15	9.80	64.46	13.73	4.90	0.49
	2079–2112	0.98	3.68	7.84	63.73	13.73	7.35	2.70
Bandar abas	2011–2044	3.43	9.80	13.97	65.93	6.37	0.25	0.25
	2045–2078	0.74	5.64	7.84	69.61	12.25	3.43	0.49
	2079–2112	1.23	6.62	7.60	63.24	13.73	4.90	2.70
Birjand	2011–2044	3.43	9.80	16.18	64.71	4.90	0.74	0.25
	2045–2078	1.23	4.41	7.60	70.83	11.76	3.92	0.25
	2079–2112	0.74	4.17	5.88	59.07	17.16	12.25	0.74
Bojnoord	2011–2044	1.72	8.82	12.50	64.95	9.56	2.45	0.00
	2045–2078	1.47	5.64	7.60	67.16	12.50	5.64	0.00
	2079–2112	0.98	5.15	10.29	58.33	15.20	8.33	1.72
Boshehr	2011–2044	3.92	6.37	14.22	64.71	8.58	2.21	0.00
	2045–2078	1.47	5.15	6.86	67.16	15.20	2.94	1.23
	2079–2112	1.72	3.68	9.31	59.80	17.65	5.64	2.21
Esfahan	2011–2044	0.00	0.74	3.43	81.13	13.48	1.23	0.00
	2045–2078	0.25	0.49	2.70	70.34	23.53	2.70	0.00
	2079–2112	2.21	16.18	52.94	25.25	1.23	0.98	1.23
Ghazvin	2011–2044	2.45	10.05	18.38	60.54	7.11	1.23	0.25
	2045–2078	0.49	4.41	11.27	68.14	12.01	3.19	0.49
	2079–2112	0.74	3.19	9.80	56.13	19.61	9.31	1.23
Ghom	2011–2044	3.43	9.31	14.46	65.44	6.13	1.23	0.00
	2045–2078	0.98	4.66	9.31	69.36	10.78	4.66	0.25
	2079–2112	0.98	5.15	5.64	60.54	16.42	9.31	1.96
Gorgan	2011–2044	2.94	5.88	12.75	66.42	8.82	2.94	0.25
	2045–2078	1.23	4.66	12.01	63.73	13.48	4.17	0.74
	2079–2112	1.47	4.17	11.76	60.05	14.71	6.37	1.47
Hamedan	2011–2044	3.68	5.88	14.95	65.93	7.60	1.72	0.25
	2045–2078	1.96	4.41	6.13	71.08	13.24	2.94	0.25
	2079–2112	1.23	4.17	7.35	56.37	22.06	5.88	2.94
Ilam	2011–2044	2.94	7.11	21.81	61.27	4.66	2.21	0.00
	2045–2078	1.47	3.68	7.11	73.28	10.29	2.94	1.23
	2079–2112	1.23	3.43	8.82	58.33	16.67	9.31	2.21
Karaj	2011–2044	2.94	9.31	16.18	62.01	7.35	2.21	0.00
	2045–2078	1.23	3.92	8.82	70.34	11.27	4.17	0.25
	2079–2112	0.49	5.88	5.15	58.82	16.91	11.03	1.72
Kerman	2011–2044	2.70	12.99	15.20	63.97	4.17	0.98	0.00
	2045–2078	0.49	3.68	8.58	70.34	12.01	4.17	0.74
	2079–2112	0.98	4.17	6.37	61.27	14.95	10.05	2.21
Kermanshah	2011–2044	3.43	7.11	19.36	61.03	7.11	1.96	0.00
	2045–2078	0.98	3.19	7.84	72.30	11.76	3.68	0.25
	2079–2112	1.23	4.66	6.62	57.35	17.89	11.03	1.23
Khoram abad	2011–2044	2.45	10.05	17.89	59.07	8.82	1.72	0.00
	2045–2078	1.23	3.68	6.37	72.55	12.25	3.68	0.25
	2079–2112	1.72	3.92	6.13	59.31	18.14	9.56	1.23
Mashhad	2011–2044	3.68	8.58	12.99	64.22	6.13	3.92	0.49
	2045–2078	1.47	2.94	9.80	69.61	12.99	2.94	0.25
	2079–2112	1.23	3.19	9.07	59.56	16.67	9.31	0.98
Oroomie	2011–2044	2.94	9.56	14.22	64.95	6.86	1.47	0.00
	2045–2078	0.98	5.64	10.54	68.38	9.80	4.17	0.49
	2079–2112	0.25	4.17	7.35	58.58	18.87	8.58	2.21
Rasht	2011–2044	2.94	6.13	12.50	63.73	10.78	3.43	0.49
	2045–2078	2.45	5.39	10.78	65.44	10.78	4.17	0.98
	2079–2112	0.49	4.17	8.09	65.69	13.73	7.35	0.49
Sanandaj	2011–2044	3.19	9.31	16.91	62.50	7.11	0.74	0.25
	2045–2078	0.74	5.88	7.11	69.12	13.97	2.45	0.74
	2079–2112	0.74	3.92	7.84	57.35	19.36	10.05	0.74
Semnan	2011–2044	2.45	10.29	13.97	66.91	5.88	0.49	0.00
	2045–2078	0.74	4.90	10.29	67.40	12.50	3.92	0.25
	2079–2112	0.74	4.90	7.60	57.84	16.42	11.27	1.23
Shahrekord	2011–2044	2.45	10.29	21.08	59.56	5.64	0.74	0.25
	2045–2078	0.49	3.68	8.33	69.12	15.69	2.21	0.49
	2079–2112	0.98	3.43	8.58	54.17	23.53	8.33	0.98
Shiraz	2011–2044	1.96	10.29	23.04	55.39	7.60	1.72	0.00
	2045–2078	0.49	2.94	7.11	72.79	12.50	4.17	0.00
	2079–2112	0.98	3.92	6.62	55.39	20.34	11.27	1.47
Tabriz	2011–2044	3.19	7.60	18.38	62.01	7.60	1.23	0.00
	2045–2078	0.98	4.66	11.52	64.71	13.24	4.66	0.25
	2079–2112	0.74	3.43	7.60	59.80	19.12	8.82	0.49
Tehran	2011–2044	2.94	8.82	12.75	65.93	7.84	1.72	0.00
	2045–2078	1.23	5.88	7.35	68.87	12.75	3.92	0.00
	2079–2112	0.74	5.15	7.11	58.33	15.20	12.25	1.23
Yasooj	2011–2044	2.45	9.07	15.20	63.97	7.84	1.47	0.00
	2045–2078	0.98	4.17	8.58	71.32	11.76	2.94	0.25
	2079–2112	1.72	4.66	8.33	56.62	19.61	7.35	1.72
Yazd	2011–2044	3.43	9.80	19.36	62.99	3.68	0.74	0.00
	2045–2078	0.74	4.90	6.86	72.55	10.78	3.19	0.98
	2079–2112	0.74	3.19	4.17	58.33	22.06	8.82	2.70
Zahedan	2011–2044	3.19	8.09	19.85	64.22	4.41	0.25	0.00
	2045–2078	1.23	3.68	8.33	72.30	12.25	0.98	1.23
	2079–2112	0.98	4.41	7.60	54.90	21.57	8.58	1.96
Zanjan	2011–2044	1.96	9.80	12.25	65.93	6.86	3.19	0.00
	2045–2078	1.72	4.17	9.56	67.65	12.01	4.66	0.25
	2079–2112	0.98	3.19	10.29	60.54	13.97	8.58	2.45
Total	2011–2044	2.85	8.47	15.40	64.40	7.15	1.64	0.09
	2045–2078	1.11	4.48	8.52	69.31	12.58	3.56	0.44
	2079–2112	1.02	4.66	9.13	57.43	17.26	8.87	1.64

**Table 5:** Frequency of the SPEI based on the percentage of months under the A1B scenario.

		Extreme wet	Severe wet	Moderate wet	Normal	Moderate drought	Severe drought	Extreme drought
Ahvaz	2011–2044	1.96	11.03	19.36	61.27	5.39	0.98	0.00
	2045–2078	0.98	4.90	4.90	78.43	8.09	2.70	0.00
	2079–2112	1.47	4.41	3.19	54.90	20.83	14.22	0.98
Arak	2011–2044	2.94	8.82	16.91	62.01	8.33	0.98	0.00
	2045–2078	0.25	5.88	7.84	71.81	12.01	2.21	0.00
	2079–2112	0.49	5.15	6.62	49.02	28.43	9.31	0.98
Ardebil	2011–2044	4.41	7.11	15.69	62.01	9.31	1.23	0.25
	2045–2078	0.98	4.90	8.58	71.57	8.58	4.41	0.98
	2079–2112	0.74	3.19	6.86	61.03	18.14	7.35	2.70
Bandar abas	2011–2044	3.19	9.56	14.46	68.38	4.17	0.25	0.00
	2045–2078	1.96	6.13	8.82	71.81	10.05	0.74	0.49
	2079–2112	0.25	5.15	4.90	59.07	20.83	8.09	1.72
Birjand	2011–2044	3.68	8.58	21.32	61.76	3.68	0.74	0.25
	2045–2078	0.25	4.17	6.86	77.94	8.82	1.72	0.25
	2079–2112	0.49	3.19	4.90	52.45	21.81	16.42	0.74
Bojnoord	2011–2044	2.21	10.05	14.22	64.22	7.84	1.47	0.00
	2045–2078	1.72	6.13	8.58	69.85	9.80	3.92	0.00
	2079–2112	0.00	2.94	6.62	57.60	19.85	10.54	2.45
Boshehr	2011–2044	3.43	7.60	16.18	63.24	8.09	1.47	0.00
	2045–2078	2.21	4.66	8.09	67.65	13.97	2.70	0.74
	2079–2112	1.72	4.17	6.37	60.29	18.14	7.11	2.21
Esfahan	2011–2044	3.43	7.84	24.26	58.82	4.90	0.74	0.00
	2045–2078	0.74	4.41	7.11	75.74	10.05	1.96	0.00
	2079–2112	1.23	3.68	6.37	49.02	26.96	10.78	1.96
Ghazvin	2011–2044	1.72	10.54	20.83	59.56	5.88	1.23	0.25
	2045–2078	0.25	4.17	11.27	72.30	9.56	1.96	0.49
	2079–2112	0.74	3.92	6.86	53.19	23.77	10.29	1.23
Ghom	2011–2044	3.43	9.31	18.38	62.75	5.39	0.74	0.00
	2045–2078	0.98	4.66	6.86	75.98	7.35	4.17	0.00
	2079–2112	1.23	3.19	5.39	54.41	22.79	11.27	1.72
Gorgan	2011–2044	2.94	6.37	12.99	65.93	8.33	3.19	0.25
	2045–2078	1.47	5.88	9.31	66.42	12.01	4.17	0.74
	2079–2112	1.47	4.17	10.05	59.80	14.46	8.58	1.47
Hamedan	2011–2044	3.68	6.13	13.24	67.65	7.35	1.72	0.25
	2045–2078	1.23	4.90	7.35	73.77	10.05	2.45	0.25
	2079–2112	1.72	3.19	6.62	51.72	26.23	9.07	1.47
Ilam	2011–2044	1.72	6.62	25.00	58.33	5.15	3.19	0.00
	2045–2078	1.72	3.68	8.33	74.75	8.58	2.21	0.74
	2079–2112	1.72	3.92	6.62	55.64	21.32	8.82	1.96
Karaj	2011–2044	2.21	9.80	18.38	62.01	6.13	1.47	0.00
	2045–2078	0.49	4.66	7.60	73.77	9.56	3.92	0.00
	2079–2112	1.23	4.17	7.11	51.47	21.81	12.50	1.72
Kerman	2011–2044	4.41	9.07	16.91	64.71	3.68	1.23	0.00
	2045–2078	0.49	3.43	8.82	77.45	7.11	2.45	0.25
	2079–2112	0.25	3.43	4.90	53.19	22.55	12.99	2.70
Kermanshah	2011–2044	2.21	6.86	22.06	59.80	7.35	1.72	0.00
	2045–2078	0.74	3.43	7.84	76.72	7.84	3.43	0.00
	2079–2112	1.72	4.66	6.13	51.47	22.55	13.24	0.25
Khoram abad	2011–2044	1.72	9.56	22.55	56.86	7.11	2.21	0.00
	2045–2078	1.23	3.43	6.86	74.51	10.78	3.19	0.00
	2079–2112	1.72	4.17	5.88	54.66	22.06	10.29	1.23
Mashhad	2011–2044	1.96	7.60	15.69	67.65	6.86	0.25	0.00
	2045–2078	1.96	4.90	7.60	73.04	7.84	4.41	0.25
	2079–2112	1.72	2.45	7.35	54.66	20.10	11.76	1.96
Oroomie	2011–2044	2.70	10.29	15.69	65.44	4.90	0.98	0.00
	2045–2078	1.23	3.92	9.07	73.53	10.05	1.96	0.25
	2079–2112	1.23	2.70	6.13	53.68	23.28	11.76	1.23
Rasht	2011–2044	2.94	6.62	11.27	63.97	10.05	4.41	0.74
	2045–2078	1.23	3.92	10.78	66.67	11.27	5.64	0.49
	2079–2112	1.23	4.41	9.80	63.48	12.50	7.60	0.98
Sanandaj	2011–2044	2.70	10.78	17.40	62.01	6.37	0.49	0.25
	2045–2078	0.74	4.17	9.31	74.75	8.33	2.21	0.49
	2079–2112	1.47	3.19	5.88	54.41	21.57	12.50	0.98
Semnan	2011–2044	3.19	10.05	14.46	65.93	6.13	0.25	0.00
	2045–2078	0.49	4.90	11.27	71.32	9.80	2.21	0.00
	2079–2112	0.74	2.45	6.86	52.21	22.30	13.48	1.96
Shahrekord	2011–2044	0.98	9.31	25.98	57.60	5.64	0.25	0.25
	2045–2078	0.49	3.92	8.33	75.00	10.05	1.72	0.49
	2079–2112	1.47	2.45	6.37	49.26	28.68	11.03	0.74
Shiraz	2011–2044	1.96	11.03	25.00	53.19	8.09	0.74	0.00
	2045–2078	0.74	3.19	6.37	77.94	8.58	3.19	0.00
	2079–2112	0.25	2.94	5.15	48.77	27.94	14.22	0.74
Tabriz	2011–2044	2.70	9.31	19.85	61.76	5.39	0.98	0.00
	2045–2078	0.98	3.68	11.27	70.10	11.52	2.45	0.00
	2079–2112	0.49	3.19	6.62	53.43	24.75	10.78	0.74
Tehran	2011–2044	2.94	9.56	14.95	63.97	6.86	1.72	0.00
	2045–2078	0.98	4.66	8.09	72.55	10.54	3.19	0.00
	2079–2112	1.23	4.66	6.13	54.17	19.36	14.22	0.25
Yasooj	2011–2044	3.19	7.84	18.63	61.76	7.11	1.47	0.00
	2045–2078	0.98	5.15	8.33	73.77	9.31	2.21	0.25
	2079–2112	0.49	4.41	6.86	48.53	29.17	9.56	0.98
Yazd	2011–2044	2.94	9.80	23.04	61.27	2.94	0.00	0.00
	2045–2078	0.74	3.43	6.86	78.19	8.09	2.70	0.00
	2079–2112	0.49	3.19	3.19	49.51	29.90	11.76	1.96
Zahedan	2011–2044	3.19	9.31	21.32	62.99	2.94	0.25	0.00
	2045–2078	0.49	5.15	8.58	77.21	7.35	1.23	0.00
	2079–2112	0.49	3.43	5.15	49.75	27.94	11.52	1.72
Zanjan	2011–2044	2.21	9.31	14.71	64.95	6.37	2.45	0.00
	2045–2078	0.98	3.68	10.54	70.34	10.29	4.17	0.00
	2079–2112	0.98	3.92	7.35	57.84	15.93	11.76	2.21
Total	2011–2044	2.76	8.86	18.36	62.39	6.26	1.29	0.08
	2045–2078	0.99	4.47	8.38	73.50	9.57	2.85	0.24
	2079–2112	1.02	3.67	6.27	53.95	22.53	11.09	1.46

**Table 6:** Frequency of the SPEI based on the percentage of months under the A2 scenario.

		Extreme wet	Severe wet	Moderate wet	Normal	Moderate drought	Severe drought	Extreme drought
Ahvaz	2011–2044	2.70	9.80	21.08	61.27	4.41	0.74	0.00
	2045–2078	1.96	4.66	6.37	79.41	6.37	1.23	0.00
	2079–2112	0.49	1.72	3.92	51.23	25.49	15.69	1.47
Arak	2011–2044	2.94	8.82	16.67	63.48	7.11	0.98	0.00
	2045–2078	0.98	6.13	9.56	72.30	9.56	1.47	0.00
	2079–2112	0.25	2.70	4.41	49.26	31.62	11.03	0.74
Ardebil	2011–2044	3.19	8.33	15.93	65.44	6.13	0.98	0.00
	2045–2078	0.98	5.64	11.52	72.30	7.11	2.45	0.00
	2079–2112	1.23	3.19	3.19	57.35	21.08	10.54	3.43
Bandar abas	2011–2044	3.43	9.80	15.20	67.89	3.43	0.00	0.25
	2045–2078	0.74	6.37	8.33	74.26	9.80	0.49	0.00
	2079–2112	1.47	3.68	6.86	52.70	22.79	9.07	3.43
Birjand	2011–2044	3.92	8.58	18.87	62.01	5.64	0.74	0.25
	2045–2078	0.98	4.41	7.35	68.87	12.99	5.15	0.25
	2079–2112	0.49	4.41	5.64	62.50	14.95	11.52	0.49
Bojnoord	2011–2044	1.47	9.56	14.22	64.95	8.09	1.72	0.00
	2045–2078	1.47	7.60	11.76	66.67	10.05	2.45	0.00
	2079–2112	0.25	2.45	5.88	58.58	20.59	9.80	2.45
Boshehr	2011–2044	4.17	8.09	16.91	62.01	7.60	1.23	0.00
	2045–2078	1.47	6.13	6.13	72.55	11.27	1.96	0.49
	2079–2112	1.23	1.47	7.11	57.60	20.83	9.31	2.45
Esfahan	2011–2044	2.94	8.33	25.00	59.07	3.92	0.74	0.00
	2045–2078	1.23	5.15	8.33	77.21	6.86	1.23	0.00
	2079–2112	0.25	1.72	4.90	45.10	34.07	12.25	1.72
Ghazvin	2011–2044	1.72	10.78	21.57	60.05	4.66	1.23	0.00
	2045–2078	0.98	5.39	10.29	74.26	7.35	1.47	0.25
	2079–2112	0.49	1.72	6.86	48.04	30.39	11.27	1.23
Ghom	2011–2044	3.92	8.58	18.38	63.73	4.90	0.49	0.00
	2045–2078	1.23	6.37	6.37	77.45	5.88	2.70	0.00
	2079–2112	0.74	1.72	4.90	47.55	31.37	12.01	1.72
Gorgan	2011–2044	2.70	8.58	11.27	66.91	7.84	2.45	0.25
	2045–2078	2.70	4.66	12.75	66.42	9.56	3.68	0.25
	2079–2112	0.98	2.21	6.86	63.48	15.69	9.07	1.72
Hamedan	2011–2044	4.17	5.64	13.97	68.38	5.88	1.72	0.25
	2045–2078	2.45	4.41	11.27	72.30	7.60	1.72	0.25
	2079–2112	0.00	2.21	4.17	50.25	30.15	12.75	0.49
Ilam	2011–2044	2.70	6.62	23.77	61.52	3.43	1.96	0.00
	2045–2078	2.45	3.19	11.27	74.02	6.62	2.45	0.00
	2079–2112	0.74	1.23	3.19	50.98	31.13	11.52	1.23
Karaj	2011–2044	2.45	8.58	19.36	62.25	5.88	1.47	0.00
	2045–2078	1.23	5.88	8.09	74.75	7.35	2.70	0.00
	2079–2112	0.49	2.70	4.66	51.96	25.49	13.73	0.98
Kerman	2011–2044	2.45	13.73	19.12	61.27	3.19	0.25	0.00
	2045–2078	0.49	4.17	7.84	77.94	7.35	2.21	0.00
	2079–2112	0.25	2.21	4.41	49.75	26.96	14.71	1.72
Kermanshah	2011–2044	2.94	7.35	22.30	61.03	5.15	1.23	0.00
	2045–2078	1.47	5.15	8.09	76.72	6.37	2.21	0.00
	2079–2112	0.49	1.72	4.41	48.28	30.64	13.97	0.49
Khoram abad	2011–2044	2.21	8.82	23.53	58.33	5.64	1.47	0.00
	2045–2078	1.47	4.90	7.84	75.49	8.33	1.96	0.00
	2079–2112	0.74	1.96	3.43	49.51	29.66	13.48	1.23
Mashhad	2011–2044	2.21	7.35	16.42	65.93	7.60	0.49	0.00
	2045–2078	2.70	6.37	9.31	69.85	6.62	4.90	0.25
	2079–2112	0.74	0.98	6.62	54.17	23.04	13.24	1.23
oroomie	2011–2044	2.45	10.05	16.67	65.20	4.90	0.74	0.00
	2045–2078	1.23	3.92	12.50	72.30	8.33	1.47	0.25
	2079–2112	0.25	2.45	4.41	50.49	28.92	11.76	1.72
Rasht	2011–2044	2.94	6.13	12.50	63.73	10.78	3.43	0.49
	2045–2078	2.45	5.39	10.78	65.44	10.78	4.17	0.98
	2079–2112	0.49	4.17	8.09	65.69	13.73	7.35	0.49
Sanandaj	2011–2044	2.70	9.80	19.12	61.76	6.13	0.49	0.00
	2045–2078	1.96	4.66	10.05	75.98	6.37	0.49	0.49
	2079–2112	0.25	2.45	3.43	50.98	27.94	13.97	0.98
Semnan	2011–2044	2.70	11.27	15.20	65.20	5.64	0.00	0.00
	2045–2078	0.74	7.11	9.80	74.26	7.60	0.49	0.00
	2079–2112	0.00	1.47	5.39	48.53	29.17	13.97	1.47
Shahrekord	2011–2044	2.21	8.82	26.72	56.62	5.15	0.25	0.25
	2045–2078	0.98	4.66	10.05	75.25	7.84	0.74	0.49
	2079–2112	0.49	1.47	3.92	44.36	36.03	13.24	0.49
Shiraz	2011–2044	1.72	10.78	26.23	53.68	6.86	0.74	0.00
	2045–2078	0.49	3.43	7.84	77.94	8.33	1.96	0.00
	2079–2112	0.25	1.47	4.41	43.87	34.56	15.20	0.25
Tabriz	2011–2044	2.94	8.33	18.87	63.24	5.64	0.98	0.00
	2045–2078	0.98	5.39	12.75	71.32	7.84	1.72	0.00
	2079–2112	0.49	1.96	5.15	50.74	27.21	13.97	0.49
Tehran	2011–2044	2.94	8.82	15.93	64.46	6.13	1.72	0.00
	2045–2078	1.23	6.86	7.35	74.51	7.35	2.70	0.00
	2079–2112	0.25	2.70	4.90	52.45	24.02	15.69	0.00
Yasooj	2011–2044	3.43	8.58	17.65	63.48	5.88	0.98	0.00
	2045–2078	1.23	5.64	8.82	74.75	8.33	1.23	0.00
	2079–2112	0.00	2.45	6.13	45.59	34.80	10.29	0.74
Yazd	2011–2044	2.70	10.29	25.74	58.58	2.70	0.00	0.00
	2045–2078	0.98	3.43	8.09	81.13	4.90	1.47	0.00
	2079–2112	0.49	1.47	3.43	43.87	35.29	13.24	2.21
Zahedan	2011–2044	3.92	7.60	24.02	62.01	2.45	0.00	0.00
	2045–2078	0.98	3.68	7.35	79.41	7.84	0.74	0.00
	2079–2112	0.74	1.72	6.13	47.06	29.66	13.24	1.47
zanjan	2011–2044	2.70	8.82	14.71	65.69	7.11	0.98	0.00
	2045–2078	0.98	6.62	12.01	71.57	6.37	2.21	0.25
	2079–2112	0.25	2.21	4.17	51.96	24.02	16.67	0.74
Total	2011–2044	2.85	8.89	18.90	62.64	5.66	1.01	0.06
	2045–2078	1.37	5.25	9.33	73.89	7.96	2.06	0.14
	2079–2112	0.51	2.20	5.03	51.46	27.04	12.45	1.31

From Table 4, it can be seen that the response of each station to future climate change is completely different during the studied period. Nevertheless, for most of the stations, the percentage of drought events in the third period (2079–2112) is more than the other periods. The average percentages of potential drought occurrences for all the stations in the next three periods are 8.89, 16.58, and 27.27 respectively. These values refer that the probability of drought will sharply increase over time. On average, about 17.74 percent of months over all the stations are likely to experience drought throughout the future years up to 2112. This finding reveals that the frequency of drought events will increase under the B1 scenario compared to the base period. Among them, the highest percentage of dry months is observed in Shiraz with 19.69%, while the lowest proportion is related to Bandarabas with 14.79 percent. Comparison of dry months demonstrates that the great number of months with extreme drought is seen in Yazd with 1.23 percent. For all stations, the number of months with moderate drought is substantially more than severe and extreme droughts. In contrast, the consequences of the humid events indicate that approximately 18.55 percent of months in all stations will experience wet months. Esfahan will experience the highest frequency of humid months over the upcoming period; however, the number of months with extreme wet is seen in Boshehr with 2.37%(29 months). It is remarkable that for all stations, the occurrence probability of moderate humid months is more than that of severe and extreme humid months. Although, the number of months with extreme wet or extreme drought is substantially less than others, it should be noted that losses and damages of extreme wet or drought would be considerably more than other situations. Thus, crisis management in each region should be designed for extreme conditions.

From the data given in Table 5 it can be inferred that like the results of Table 4, for all the regions the frequency of drought in the third period is substantially more than the previous periods. This value is about 35.08 percent, which is higher than the B1 scenario, by a broad margin. As an overall trend, the results of Table 5 indicate that, the frequency of dry months in Iran over the studied period (from 2011 to 2112) will be about 18.46 percent, which is more than the observations in the B1 scenario (e.g., 17.74 percent). Thus, it can be concluded that the proportion of predicted drought under the A1B scenario is more than the B1 scenario. Subsequently, this value is far more than the number of droughts in the base period. This finding is in agreement with the survey of [43], who reported that the emission scenario of B1 always produces less severe extreme disasters. This is because the emission scenario of B1 assumes that the future world will be sustainable with quick variations in economic structures and more efforts in introducing clean technologies.

Detailed consequences illustrate that the number of months that will be affected by moderate drought is considerably more than others. Arak can be introduced as a station that will face the highest number of moderate droughts over the next period, with 199 cases (16.26%). However, the most proportion of months with extreme drought will be seen in Ardebil with 1.31percent, which can be a serious problem for water resources of this region. Overall, the highest frequency of dry months will belong to Shiraz, 259 months from 1224 months (21.16%). Thus, it can be mentioned that the probability of drought incidence in Shiraz would be the most among all the stations.

Evaluating the results related to humid periods depict that for all stations, the number of months with moderate wet is significantly more than those with severe and extreme wet. Moreover, the number of months with extreme wet is considerably less than the severe wet. Looking in more detail, it can be seen that the highest frequency of humid months will be observed in Ghazvin with 20.10 percent, whereas the lowest frequency would be related to Hamedan, with 16.01percent. The findings of wet intensity show that the greatest number of extreme wets will occur in Boshehr, with 2.46 percent in comparison to 0.90% for Ghazvin as the lowest number.

The information given in Table 6 depicts that the frequency of droughts in the third period are dramatically more than the previous periods. This difference is completely significant. This amount is about 40.81 percent in comparison to 6.72 and 10.16percent for the first and second periods respectively.

It can be seen from Table 6 that the number of dry months under the A2 scenario (19.23percent) is more than the B1 and A1B scenarios (17.74 and 18.46percent). Due to the fact that the A2 scenario is introduced as a high emission scenario which describes a very heterogeneous world, this result seems to be reasonable and rational. Furthermore, this finding is consistent with other studies. For instance, in the survey of Akbari *et al*. (2016) [44], the highest temperature, and the lowest precipitation prognosticated belonged to the A2 scenario. Furthermore, this result corresponds to the results of [45]. They indicated that a drought event with more intensity, duration and frequency than the base period can occur in future periods under the A2 scenario in northwest of Iran. According to the study of Modarres *et al*. (2018) [46], the average of the rainfall events will reduce in evaluating future extreme rainfall changes in the north of Iran as well. It can be an alarm in applying effective strategies for the future water resources in this area. An increasing trend of drought under the A2 scenario has been declared by [47], which is in line with the present forecasted consequences. The conducted research by [48] for assessing the future temperature in Iran, researchers declared that the maximum temperature changes will be increased more severely based on the A2 scenario compared to other scenarios, which is in line with the current investigation. Although in some investigations like [21], an increased precipitation under the B1 scenario compared to other scenarios has been reported, it should be mentioned that the time scale is different in this study. Moreover, since the changes of both precipitation and PET are taken into account for calculating SPEI, the interpretation of SPEI can be distinct from precipitation.

In parallel with results of the B1 and A1B scenarios, the most frequency of dry months is observed in Shiraz with 22.63percent, while this value for the B1 and A1B scenarios are 19.70 and 21.16 percent (Tables 4 and 5). 1.23percent of months in Bandarabbas expected to experience extreme droughts, which can be labeled as the driest station throughout the next years. This result matches with the survey of [37]. Due to the proportion of dry months, it can be inferred that approximately over 16.42 percent of months of each station will be experiencing droughts during the next years, which is alarming for Iran. Although the number of months with extreme droughts is much more than other categories, it should be declared that losses and damages of extreme intensity droughts are dramatically more destructive than others. In other words, a short-time drought period with extreme intensity can have long-term repercussions.

The highest figure of humid months under the A2 scenario belongs to Ghazvin with 19.93percent, which is similar to the results of the A1B scenario. It is prominent that for all the stations, the number of months with moderate wet is significantly more than the cumulative amount of extreme and severe wets. The highest number of months with extreme wet is related to Bosheher with 2.30 percent.

**Fig 4:**
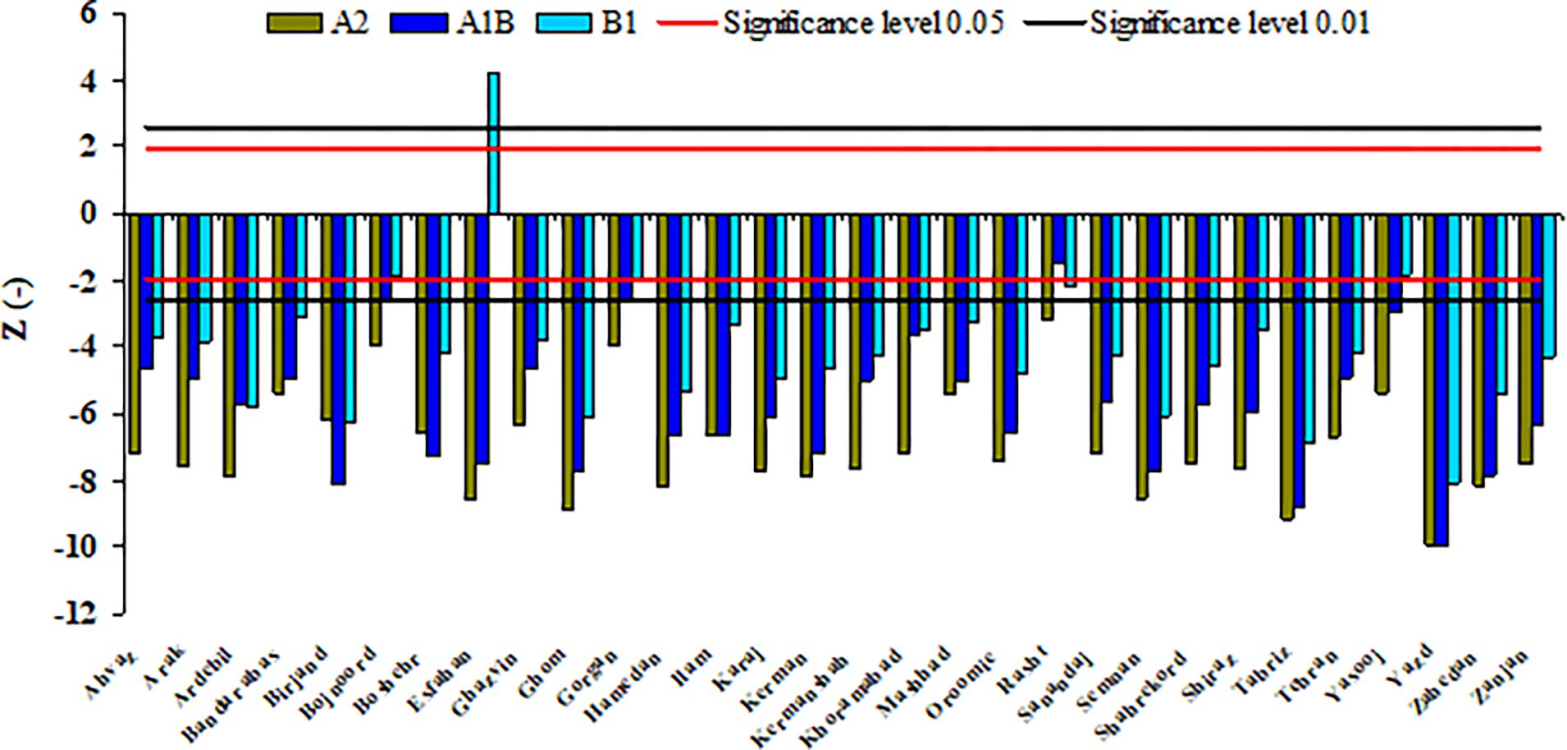
The result of Mann-Kendall test on SPEI trend over the whole period.

For examining the changes in the trend of SPEI over the studied period from 2011 to 2112, the results of Mann-Kendall test (Z) are provided in Fig 4. The aim of this test is to statistically evaluate if there is a monotonic increase or decrease trend of the studied variables over the time. It is noticeable that *Z*≥|1.96| refers to the significance level 0.05, while *Z*≥|2.58| implies to the significance level 0.01.

Fig 4 indicates that there is a downward trend in the SPEI changes in all the stations, except Esfahan under the B1 scenario. In other words, the value of SPEI over time will decrease significantly. So that, for most of the stations, this trend is significant in the level of 0.01. The highest downward trend for a large number of stations is observed in the A2 scenario. Thus, it can be declared that the amount of SPEI will drop sharply over time and this decrease under the A2 scenario will be the most intensive.

As a consequence, it can be inferred that the A2 scenario predicts the most serious drought in comparison to other scenarios. Moreover, since the survey of [36] demonstrated that the certainty of the predicted PET values under the A2 scenario is higher than the other scenarios, the results of the A2 scenario were examined for extracting the characteristics of SPEI based on the Run theory,. Findings are provided in Table 7 for each station for the next 102 years.

**Table 7:** Characteristics of dry and wet periods based on SPEI under the A2 scenario.

Station	Dry period				Wet period			
	Number of periods	Longest period	Maximum deficit	Total deficit	Number of periods	Longest period	Maximum surplus	Total surplus
Ahvaz	210	24	22.73	501.43	211	15	14.55	508.67
Arak	246	20	24.43	506.77	252	14	12.28	512.99
Ardebil	271	15	16.1	497.68	268	8	11.	504.91
Bandar abas	266	16	17.7	488.75	263	19	16.4	503.50
Birjand	250	18	17.26	500.57	251	15	14.81	508.13
Bojnoord	269	16	14.84	504.92	264	8	9.8	510.39
Boshehr	281	14	12.06	494.97	275	17	19.36	505.15
Esfahan	217	21	21.01	506.28	222	15	16.99	512.71
Ghazvin	216	21	19.53	514.54	223	12	13.14	519.73
Ghom	232	22	22.69	503.45	240	16	16.86	509.59
Gorgan	286	8	8.50	504.00	285	8	9.67	510.38
Hamedan	253	22	21.58	507.41	252	12	14.37	512.47
Ilam	233	17	19.34	504.62	231	20	20.41	510.20
Karaj	258	10	11.12	507.75	260	12	12.78	512.45
Kerman	216	19	22.02	505.28	224	31	30.31	510.99
Kermanshah	234	16	17.41	506.19	237	10	11.51	511.46
Khoramabad	231	19	16.38	511.63	241	18	20.71	514.76
Mashhad	231	25	21.52	503.20	244	14	13.11	508.52
Oroomie	253	19	16.03	500.67	246	18	14.98	507.71
Rasht	303	19	17.24	502.56	309	6	6.77	509.73
Sanandaj	225	14	14.72	506.03	228	15	11.82	513.01
Semnan	228	14	13.71	504.03	232	20	20.01	510.36
Shahrekord	232	24	13.87	508.38	245	23	21.94	514.23
Shiraz	229	18	19.18	512.50	225	26	24.86	515.82
Tabriz	274	10	12.07	510.63	265	12	10.53	516.59
Tehran	242	20	24.93 (	505.32	247	10	11.3	510.95
Yasooj	242	12	12.12	507.48	240	19	16.07	515.50
Yazd	225	22	23.04	504.55	227	22	21.71	508.28
Zahedan	218	25	27.36	507.67	216	20	16.97	513.33
Zanjan	241	21	23.60	501.38	242	11	11.70	506.53

As an overall trend, comparing the number of dry periods for all the stations indicate that the proportion of dry periods in humid and cold regions is more than warm climate. For instance, 288 dry months are expected for Gorgan, in comparison to 218 dry months for Zahedan. However, in regard to long dry periods, the longest dry periods are expected to be observed in warmer stations; 8 in Gorgan compared to 25 in Zahedan. The maximum deficit can also be seen in warm climates such as Zahedan, with 27.36. This consequence can be seen in other stations such as Ghom with a hot climate and Tabriz with a cold climate. In regard to wet periods, the results are the same, i.e. the highest frequency of wet periods is related to cold climates, whereas the lowest belongs to warm climates. However, the duration of dry periods in warm climates is longer than that of cold climates. The information given for Rasht and Gorgan in comparison with Zahedan and Ahvaz can confirm these declarations. Sobhan *et al*. (2019) also reported that the southern stations of Iran are more exposed to drought than others.

It should be mentioned that uncertainty in the prediction of future events is inevitable, it is significantly affected by the type of data used in terms of quality and quantity aspects, and also the structure of downscaling model [36, 49]. Regarding the data, using dataset with no missing data will reduce uncertainty, which was done in this study. Apart from that, the SPEI index assimilates more factors to quantify drought characteristics which affects less uncertainty [25]. From aspect of model, considering the calibration results, it can be claimed that the present model could discover connections of weather parameters with high accuracy, thus the performance of the model is acceptable. Moreover, different scenarios provided a chance to consider different assumptions in prediction which was compared. There is no hesitation that removal of uncertainty in modelling is impossible, nevertheless, the above-mentioned reasons can confirm reliability of the findings in this study.

**Fig 5:**
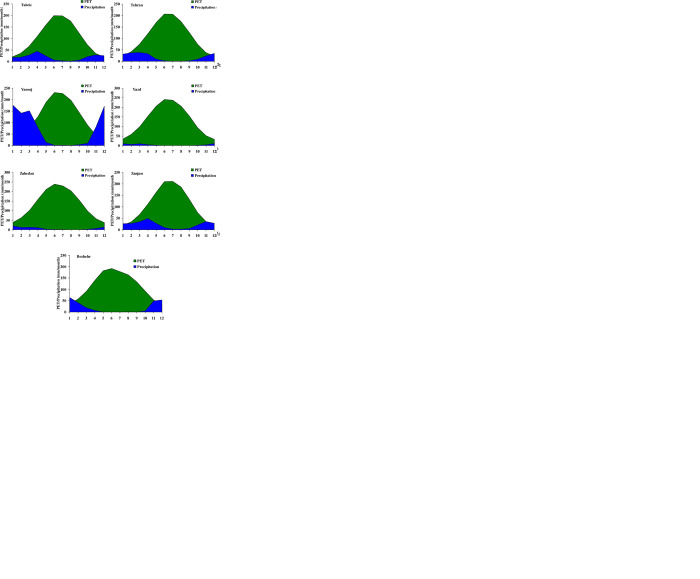
The temporal distribution of PET and precipitation for the next 102 years.

For more understanding, the monthly changes of PET and precipitation predicted under the A2 scenario for each station are provided in Fig 5. Fig 5 presents the area charts of PET and precipitation in a monthly time scale for the next 102 years.

It seems to be crystal clear that for the vast majority of stations, the area of PET is significantly more than the area of precipitation. The most difference can be seen in the middle months of the year (e.g., in warm months), which is a logical and rational consequence. These findings are consistent with the report of [50], who concluded that the magnitudes of PET in Iran increases from January to July, then drop from August to December, and all stations reach their maximum levels in July. Since the highest temperature takes place in warm months, subsequently the amount of PET would increase as warming accelerates. However, the probability of precipitation occurrence would drop. These findings can be observed in regions with hot climates such as Semnan, Yazd, Zahedan, and Kerman which are located in the southern part of Iran. [37] also declared that the southern regions of Iran are more severely affected by drought. These results refer to the importance of suitable water management in warm months and especially preserving and harvesting precipitation during the months with high precipitation. Contrary to these regions, the amount of precipitation (110.80 mm/month) in Rasht is approximately 26 percent more than the PET (87.51 mm/month). These consequences are in line with the findings of [51].

In general, it can be concluded that water shortage in warm climates would be much more serious than in other climates. Despite insufficient precipitation being the principal cause of water scarcity and drought, non-meteorological drivers such as rapid population growth, poor water resource management, and unsuitable cropping patterns also play critical roles [51].

## 4. Conclusion

Evaluating the amount of precipitation and PET for the base period and the next 102 years depicted that the highest PET and the lowest precipitation are related to the southeast of the studied area. Prediction of drought by SPEI under climate change scenarios indicated that the vast majority of stations will be involved in drought events. However, regions with hotter climates will experience more intensive drought. Comparing forecasted drought under the A2, A1B and B1 scenarios showed that 19.23% of months over the next 102 years under the A2 scenario will struggle with water shortage and dry periods, which is more than other scenarios and the base time scale. The study highlights these findings: the number of dry periods in cold and cool terrains would be more than hot climates, whereas their intensity would be less than hot and warm climates. Furthermore, the temporal distribution of PET and precipitation revealed that for most of the stations, the precipitation was considerably less than the PET, especially in the middle months of the year. This situation in the southern regions is likely to be more significant. As a consequence, the severity and number of future droughts are expected to deteriorate particularly in warmer climates in terms of both meteorological and hydrological aspects, which highlights the importance of current water resources management for transmitting these natural resources to the next generations. According to the findings, this study broadens the knowledge on the future drought condition over Iran with acute water shortage crisis. Based on the observation, different regions will encounter distinct drought circumstances in the future which necessitates implementing specific plans to address water deficit in each area.
