# Correction: Drought trends projection under future climate change scenarios for Iran region

**DOI:** 10.1371/journal.pone.0315634

**Published:** 2024-12-09

**Authors:** Maryam Bayatvarkeshi, Monzur Alam Imteaz, Ozgur Kisi, Mohammad Farahani, Mohammad Ghabaei, Ahmed Mohammed Sami Al-Janabi, Bassim Mohammed Hashim, Baqer Al-Ramadan, Zaher Mundher Yaseen

The first author’s name is spelled incorrectly. The correct name is: Maryam Bayatvarkeshi. The correct citation is: Bayatvarkeshi M, Imteaz MA, Kisi O, Farahani M, Ghabaei M, Al-Janabi AMS, et al. (2023) Drought trends projection under future climate change scenarios for Iran region. PLOS ONE 18(11): e0290698. https://doi.org/10.1371/journal.pone.0290698.

## References

[1] Bayatavrkeshi M, Imteaz MA, Kisi O, Farahani M, Ghabaei M, Al-Janabi AMS, et al. (2023) Drought trends projection under future climate change scenarios for Iran region. PLOS ONE 18(11): e0290698. 10.1371/journal.pone.0290698 37943868 PMC10635549

